# Supplementary figures and images for: Reverse Engineering Cancer: Inferring Transcriptional Gene Signatures from Copy Number Aberrations with ICAro
Source: Cancers (Basel). 2019 Feb 22;11(2):256. doi: 10.3390/cancers11020256 (PMC6406408; doi:10.3390/cancers11020256)

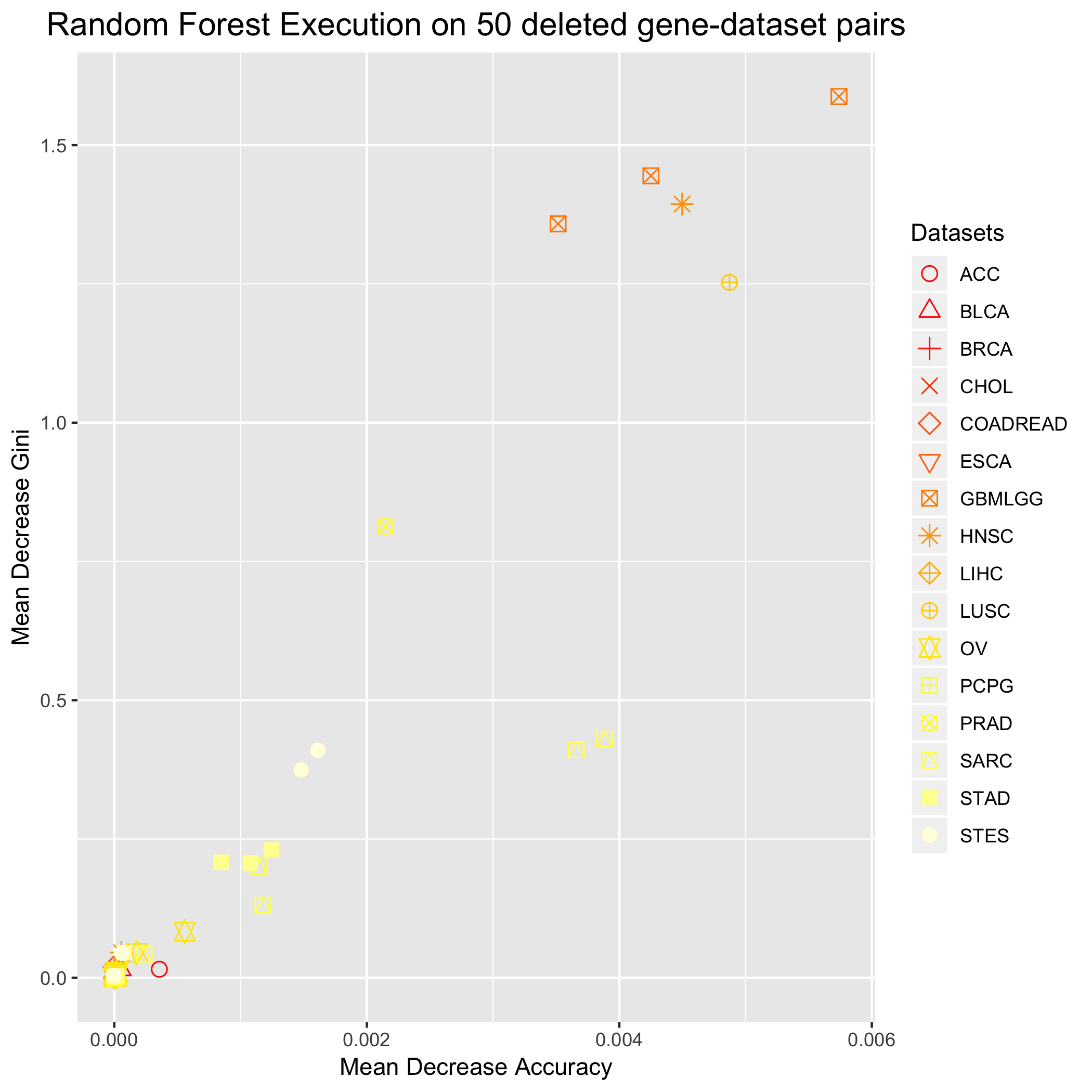

Supplement: Supplementary file 1 [file cancers-11-00256-s001.zip › FigS1.png]
